# Tissue Extracellular Matrix Nanoparticle Presentation in Electrospun Nanofibers

**DOI:** 10.1155/2014/469120

**Published:** 2014-05-29

**Authors:** Matt Gibson, Vince Beachley, Jeannine Coburn, Pierre Alain Bandinelli, Hai-Quan Mao, Jennifer Elisseeff

**Affiliations:** ^1^Translational Tissue Engineering Center and Wilmer Eye Institute, Johns Hopkins University, Baltimore, MD 21287, USA; ^2^Department of Biomedical Engineering, Johns Hopkins University, Baltimore, MD 21287, USA; ^3^Department of Materials Science and Engineering, Johns Hopkins University, Baltimore, MD 21287, USA

## Abstract

Biomaterials derived from the decellularization of mature tissues retain biological and architectural features that profoundly influence cellular activity. However, the clinical utility of such materials remains limited as the shape and physical properties are difficult to control. In contrast, scaffolds based on synthetic polymers can be engineered to exhibit specific physical properties, yet often suffer from limited biological functionality. This study characterizes composite materials that present decellularized extracellular matrix (DECM) particles in combination with synthetic nanofibers and examines the ability of these materials to influence stem cell differentiation. Mechanical processing of decellularized tissues yielded particles with diameters ranging from 71 to 334 nm. Nanofiber scaffolds containing up to 10% DECM particles (wt/wt) derived from six different tissues were engineered and evaluated to confirm DECM particle incorporation and to measure bioactivity. Scaffolds containing bone, cartilage, and fat promoted osteogenesis at 1 and 3 weeks compared to controls. In contrast, spleen and lung DECM significantly reduced osteogenic outcomes compared to controls. These findings highlight the potential to incorporate appropriate source DECM nanoparticles within nanofiber composites to design a scaffold with bioactivity targeted to specific applications.

## 1. Introduction

Naturally derived scaffolds from extracellular matrix (ECM) of human and animal tissues have received much attention for their potential application in regenerative medicine. The ECM contains an inherent biochemical and architectural complexity that influences cellular behavior and facilitates tissue development.* In vitro* studies have indicated that ECM materials retain the ability to modulate a wide variety of cell functions including gene expression, differentiation, proliferation, and migration [1–4]. Furthermore, specific cell responses were observed to be different on ECM materials from different tissues of origin and were connected to the unique biochemical and architectural makeup of each type of ECM [2, 4]. For example, sinusoidal endothelial cells were able to maintain a differentiated phenotype when cultured on ECM derived from the liver but not on ECM derived from the small intestine or bladder [4]. Removal of cells, or decellularization processing, allows for allogeneic and xenogenic clinical implantation of ECM. In addition to removing cells, common decellularization treatments such as acid, detergent, enzyme, or mechanical treatments may alter the biochemical and architectural make of the ECM [5]. Nevertheless, the remaining matrix after processing has a biomolecular complexity far exceeding that of synthetic and purified polymers and decellularized ECM (DECM) scaffolds have demonstrated efficacy in both large animal and human clinical trials [6–9]. The most significant limitation of DECM scaffolds involves material properties, such as size, shape, degradation rate, physical form, or mechanical strength. These limitations are intrinsically defined by the tissue source; an attractive alternative to overcome them is through forming composite materials with synthetic polymers.

Although lacking in biochemical complexity, the material characteristics of synthetic polymers are highly customizable. Polymer nanofibers are a promising vehicle to deliver ECM as they recapitulate the scale and structural features common to biological tissue. Nanofibers of many different types of polymer materials are easily fabricated using the electrospinning technique. The mechanical properties and physical form of electrospun nanofibers are highly customizable and biomolecules can be included to improve bioactivity. Purified biological materials, such as collagen, growth factor cocktails, or mixtures of synthetic peptides, have been combined with electrospun polymer nanofibers to mimic the natural ECM environment. These approaches have achieved some success and demonstrated superior bioactivity when compared to fully synthetic scaffolds [10, 11]. However, this strategy is limited by the difficulty of identifying optimal amounts and combinations of defined factors, preserving their biological functionality and releasing them according to suitable kinetic profiles. Recent* in vitro* studies comparing cell interactions with combinations of purified ECM proteins suggest that ECM regulatory function on cell fate is quite sensitive to the specific composition and ratio of proteins contained in the purified biological substrates [12, 13]. Furthermore, there is evidence that therapeutic biomolecules are more effective when they are bound to ECM in a natural state [14]. Such findings, taken together with the recognized complexity of natural ECM, make it seem unlikely that the functionality of ECM can be recapitulated by a limited set of synthetic or purified components.

In the present work, DECM nanoparticles from different tissues of origin were directly loaded into an electrospinning solution to fabricate polycaprolactone/DECM composite nanofiber scaffolds that combine the versatility of synthetic materials and the biological complexity of natural ECM. The bioactivity of composite scaffolds containing DECM derived from several different tissues of origin is measured by comparing their osteogenic potential* in vitro*. Our goal is to develop a modular platform where different types of source DECM can be interchangeably incorporated into a synthetic scaffold. We hypothesize that these biosynthetic scaffolds could be useful in a wide variety of applications due to the variable bioactivity of the natural DECM component and the physical tunability of the synthetic polymer component.

## 2. Materials and Methods

### 2.1. Overview of Experimental Design

Porcine ECM was isolated from the indicated organs, cryogenically pulverized, and subjected to high-pressure homogenization. Following filtration to remove aggregates and particles larger than 1.0 *μ*m, the ECM was incorporated into electrospun poly-*ε*-caprolactone (PCL) nanofibers. The resulting fibers were characterized and evaluated for osteoinductive capabilities on human adipose-derived stem cells (hASCs). PCL fibers of the same diameter were used as negative controls. Chemicals were obtained from Sigma-Aldrich (St. Louis, MO) and used as supplied, unless otherwise indicated.

### 2.2. Decellularized ECM Nanoparticle Preparation

#### 2.2.1. Tissue Decellularization

**Figure 1:**
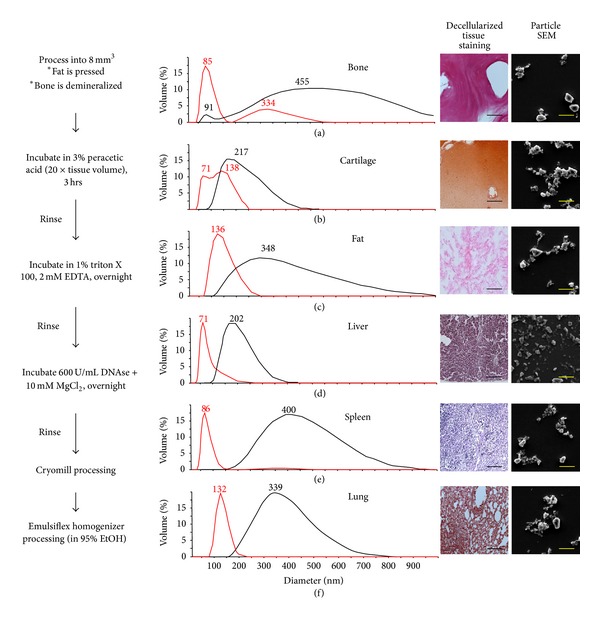
Left: processing steps used to fabricate DECM nanoparticles from whole tissue. Right: decellularized ECM particle size distributions, decellularized tissue morphology, and SEM evaluation of tissue nanoparticles. Emulsion sheering provides a narrower size distribution and limits differences in particle morphology, as demonstrated by dynamic light scattering before (black) and after (red) emulsion sheering. Histology scale bar = 50 *μ*m and SEM scale bar = 100 nm.

The tissue processing algorithm is depicted schematically in Figure 1. The indicated organs were obtained from 6-month-old market weight pigs, weighing approximately 100 kg (Wagner's Meats, Mt. Airy, MD) with approval from the Johns Hopkins Medicine Institutional Review Board. Tissue samples were obtained immediately after sacrifice and transported at 4°C to minimize degradation. Surrounding fascia and tissues were removed and the whole organ was rinsed in phosphate buffered saline (PBS) containing 100 U/mL penicillin and 10 mg/L streptomycin (1% P/S, Invitrogen, Grand Island, NY). Unless otherwise indicated, rinses and incubations throughout the decellularization process utilized a tissue sample : fluid ratio of at least 1 : 10. Each organ was treated identically with the exception of cortical long bone and adipose tissue, which required preliminary steps for demineralization and the removal of lipids, respectively. The femoral diaphysis was sawed into 10 mm cortical rings using a water-cooled band saw, decalcified with 10% formic acid for 18 hrs at room temperature (RT), and extensively rinsed in PBS. Subcutaneous adipose tissue was mechanically pressed at 37°C to reduce lipid content.

Tissue samples were cut into uniform 8 mm cubes and incubated with 3% peracetic acid for 3 hrs at 37°C. Subsequent PBS rinses facilitated neutralization and removal of the acid. The samples were then incubated in 1% triton X 100 containing 2 mM EDTA for 18 hrs at 37°C and then rinsed in PBS. The final decellularization step consisted of incubation with 600 U/mL DNAse containing 10 mM MgCl_2_ for 18 hrs at 37°C and was followed by rinsing in PBS. Decellularization was confirmed based on the DNA content and hematoxylin and eosin (H&E) staining, as described below. The ECM samples were lyophilized and stored at −20°C under argon until use. For histological analysis of decellularized ECM, samples were fixed in 10% formalin for 18 hrs at 4°C, dehydrated with ethanolic gradients, and embedded in paraffin per standard laboratory procedures. Tissue samples were collected as 7 *μ*m thick sections and stained with H&E using standard techniques.

#### 2.2.2. Nanoparticle Formation

Lyophilized ECM samples were cryogenically pulverized at −195°C under liquid N_2_ using a SPEX 6770 Freezer/Mill (SPEX SamplePrep, Metuchen, NJ) according to the manufacturer's instructions. The resulting powder was resuspended in anhydrous ethanol (ETOH) and homogenized at 170 MPa using an EmulsiFlex C3 with an in-line heat exchanger (Avestin, Inc., Ottawa, ON, Canada). The suspension was filtered to exclude particles greater than 1 *μ*m in diameter. Filtered ECM particles were introduced into anhydrous dimethyl sulfoxide (DMSO) and the ethanol was removed at room temperature using a high vacuum.

#### 2.2.3. DECM Nanoparticle Characterization

Particle size distributions were determined using a Zetasizer ZS90 (Malvern Instruments Inc., Westborough, MA) and confirmed with scanning electron microscope (SEM), as described below. Samples were sputter-coated with 10 nm of platinum (Hummer 6.2 Sputter System, Anatech USA, Union City, CA). Particle and nanofiber morphology were evaluated using a Quanta 200 SEM (FEI, Hillsboro, OR) with an acceleration voltage of 10 kV and sized using ImageJ software (National Institutes of Health, Bethesda, MD).

### 2.3. Biosynthetic ECM-Nanofiber Composites

#### 2.3.1. Electrospinning

Electrospinning was performed using a typical setup. Polymer solutions were prepared by dissolving 10% (w/v) PCL (M_*n*_ 80,000) in 9 : 1 dichloromethane (DCM) : dimethyl sulfoxide (DMSO) with the indicated amount of ECM suspended within the DMSO. For example, to prepare fibers containing 10% bone ECM, 1.0 g of PCL dissolved in 9 mL DCM was added to 0.1 g bone ECM suspended in DMSO. The resulting mixtures were sonicated for 15 sec with an output power of 7 W with an ultrasonic processor (GE 130PB, Cole Parmer) and loaded into a glass syringe for electrospinning. Solutions containing 10% ECM were prepared with bone, cartilage, fat, liver, spleen, and lung tissue. Additionally, solutions containing 1% ECM were prepared with bone and cartilage to measure dose response effects. Bone and cartilage were chosen for dose response studies because we are interested in osteogenic responses, which are most closely associated with bone and cartilage tissue in physiological bone development. The electrospinning parameters were the same for all groups. Polymer solutions were delivered via a programmable syringe pump (KD Scientific, Holliston, MA) at 0.5 mL/min through a 22 G blunt needle. A 25 kV potential was applied to the needle tip using an ES30 power supply (Gamma High Voltage Research, Ormond Beach, FL). Samples were collected onto a grounded aluminum plate covered with uniformly spaced 5 mm glass coverslips with a tip-to-collector distance of 15 cm. Fibers were secured to the underlying glass with 0.5 *μ*L drops of cyanoacrylate and removed from the surrounding fibers with a 6 mm biopsy punch. Fiber/ECM constructs were stored under vacuum at −80°C until use. They were washed with sterile PBS containing 1% P/S before cell seeding.

#### 2.3.2. Morphology Characterization

Samples were fixed in 100 mM sodium cacodylate containing 1.5% glutaraldehyde, 3% paraformaldehyde (PFA), and 2.5% sucrose for 45 min at RT. Samples were serially dehydrated through 100% ETOH, dried under high vacuum, and sputter-coated with 10 nm of platinum. Nanofiber morphology was characterized with SEM, as described above. A minimum of 200 individual fibers was counted for fiber diameter measurements. Nanofiber composites containing cartilage particles were stained with Safranin O to identify glycosaminoglycans (GAGs) in cartilage particles embedded in PCL nanofibers. Safranin O solution (100 mg Safranin-O/100 mL deionized water (dH_2_O)) was adjusted to pH 3.1 and nanofiber scaffolds were incubated for 10 min and then washed 3 times in dH_2_O.

#### 2.3.3. Contact Angle Measurements

The wettability of the electrospun fiber mats was determined using the sessile drop method. For this analysis, fiber meshes were collected onto square 1 cm glass coverslips, washed 3X with deionized water, and dried under house vacuum at RT. Droplets contained 10 *μ*L of dH_2_O and were imaged with a Discovery V8 Dissection Scope (Zeiss, Jena, Germany). Contact angles were evaluated using the code developed by Brugnara within ImageJ (NIH).

#### 2.3.4. Protein Release Studies

The* in vitro* release of protein was studied for 21 days through incubating 30–35 mg of nanofibers in 5 mL of PBS in a 15 mL conical tube. The samples were incubated at 37°C with gentle agitation and 500 *μ*L aliquots of PBS were collected, flash-frozen, and lyophilized at desired time points. Following each collection, the same volume of fresh PBS was added to maintain a consistent volume. The released protein was quantified with the QuantiPro BCA assay kit (Sigma) through measuring the absorbance at 560 nm with a NanoDrop 2000 spectrophotometer (Thermo Scientific, Wilmington, DE). Released proteins were analyzed at two dilutions and in triplicate using a 40 *μ*L reaction volume.

### 2.4. Stem Cell Culture on Nanofiber Composites

#### 2.4.1. Cell Culture and Media Conditions

hASCs were isolated and characterized, as previously described by Mitchell et al. [15], and were obtained via a material transfer agreement. The cells were passaged via trypsin digestion at 90% confluence (Gibco, Grand Island, NY) and expanded to passage 4 in medium consisting of low glucose (1.0 g/L) Dulbecco's modified Eagle medium (DMEM) supplemented with 876 mg/L of L-glutamine, 10% fetal bovine serum (FBS), 1 *μ*g/L basic fibroblast growth factor (Invitrogen, Grand Island, NY), and 1% penicillin streptomycin. Fibrous scaffolds were seeded at 5,000 cells/cm^2^ in osteogenic media composed of high glucose (4.5 g/L) DMEM supplemented with 10% FBS, 50 *μ*M ascorbic acid, 0.1 *μ*M dexamethasone, 10 mM glycerol-2-phosphate disodium salt, and 1% P/S. Cellular constructs were harvested and analyzed at the indicated time points of 3 days, 1 week, or 3 weeks.

#### 2.4.2. Cell Viability and Proliferation

Cells were seeded, as shown above, and stained after 3 days of culture using the live/dead viability/cytotoxicity kit for mammalian cells according to the manufacturer's instructions (Invitrogen, Grand Island, NY). Briefly, constructs were incubated for 30 min in DMEM containing 4 *μ*M calcein-AM and 4 *μ*M ethidium homodimer-1 at 37°C in a humidified environment containing 5% CO_2_. Samples were rinsed with warmed PBS and imaged using the appropriate fluorescent filters on an Axiovert A2 (Zeiss, Jena, Germany).

At the desired time points, constructs were washed with PBS and fixed with aqueous 4% PFA (v/v) at RT for 15 min. Cells were permeabilized with a 5 min RT incubation in 0.1% triton X 100 and then rinsed in PBS. Actin cytoskeletal staining was performed through incubating cells in 2.5% (v/v) Texas Red-X phalloidin (Invitrogen, Grand Island, NY) containing 4 *μ*M Hoechst 33258, per the manufacturer's instructions. Imaging was performed, as shown above, using the appropriate fluorescent filters; overlays were created using ImageJ software.

#### 2.4.3. Osteogenic Differentiation of hASCs

Calcified ECM production by hASCs in culture was evaluated with alizarin red staining after incubation in osteogenic media for 3 days, 1 week, or 3 weeks. Cell-laden constructs were rinsed in PBS and fixed with 4% PFA, as shown above. Subsequently, each 5 mm construct was immersed in 500 *μ*L of 40 mM alizarin red S solution (pH 4.1) for 20 min and then rinsed 3X with PBS and immediately imaged with a Zeiss Discovery v8 dissection scope. The percent area stained was quantified with ImageJ software using the threshold color function in RGB color space with pass thresholds set at red: 0–255, green: 0–104, and blue: 0–64.

Total RNA was extracted from cultured hASCs using the commercially available TRIzol reagent (Invitrogen). Briefly, two 5 mm nanofiber constructs were homogenized in 500 *μ*L TRIzol and the RNA was extracted using 500 *μ*L chloroform. RNA was precipitated using isopropanol and washed 3X in 75% ETOH. The resulting pellets were resuspended in diethylpyrocarbonate-treated water (Invitrogen, Grand Island, NY) and the RNA concentration and quality were evaluated with absorbance spectroscopy based on the absorbance at 260 and 280 nm using a NanoDrop 2000 spectrophotometer (Thermo Scientific, Waltham, MA). Cloned complementary DNA (cDNA) was synthesized by reverse transcription using the Superscript First-Strand Synthesis System (Invitrogen, Grand Island, NY) according to the manufacturer's instructions. Quantitative real-time polymerase chain reaction (RT-PCR) was performed on a StepOnePlus Real-Time PCR System using StepOne reagents with SYBR Green Master Mix, according to the manufacturer's instructions (Applied Biosystems, Grand Island, NY). Biological replicates were analyzed in triplicate with gene expression normalized to *β*-actin and fold change determined using the ΔΔC_*t*_ method. PCR primers used were RunX2 (F: CTTCACAAATCCTCCCCAAGTAGCTACC, R: GGTTTAGAGTCATCAAGCTTCTGTCTGTG), collagen I (F: GCCAAGAGGAAGGCCAAGTC, R: AGGGCTCGGGTTTCCACAC), and osteocalcin (F: CGGTGCAGAGTCCAGCAAAG, R: CTCCCAGCCATTGATACAGGTAGC).

## 3. Results

### 3.1. Decellularized ECM Nanoparticles

Combined decellularization and particulization procedures allowed the formation of DECM nanoparticles with a uniform size distribution (±~100 nm) and morphology for six tissues of varied composition and mechanical properties. Porcine-derived bone, cartilage, fat, liver, spleen, and lung tissue were decellularized, as described. Histological sections stained with H&E in Figure 1 show the tissue microstructure after decellularization and confirm the successful removal of cells from the tissues. Size distributions of the DECM particles of all types are shown after cryomill and emulsion sheering processing steps. The range of particle diameters for different tissues after cryomilling was as high as 1 *μ*m, with an overall average particle diameter of ~325 nm. Emulsion shearing considerably lowered the average particle diameter and narrowed the diameter distribution of all particle types (average diameter ~100 nm). The size and morphology of particles of each tissue type after complete processing are shown in SEM images in Figure 1.

### 3.2. Biosynthetic ECM-Nanofiber Composites

#### 3.2.1. Fiber Morphology

**Figure 2:**
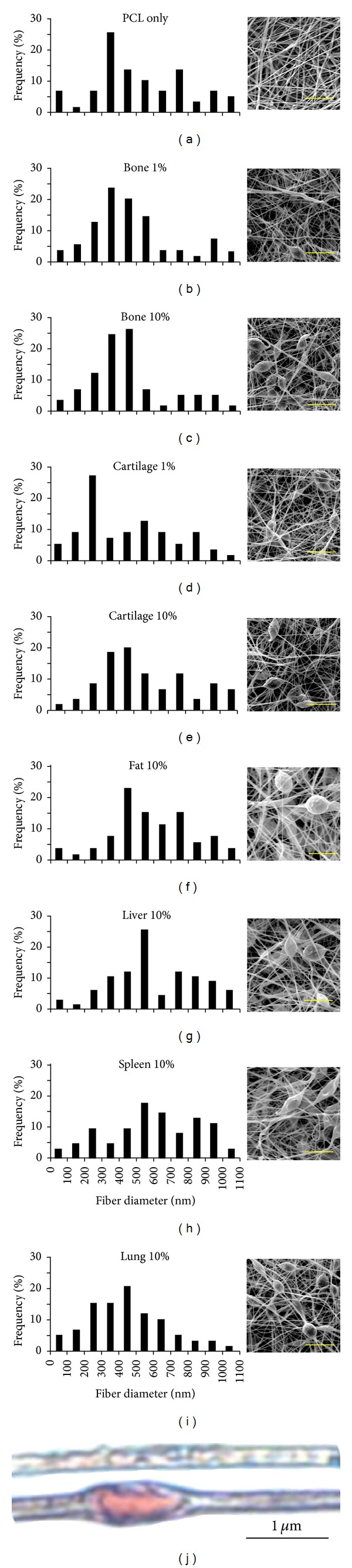
Nanofiber size distributions and morphology as seen with SEM images (a)–(i). Cartilage matrix incorporation into PCL nanofibers is visualized with Saffron O staining (pink) for glycosaminoglycans: (a) PCL only and (e) cartilage 10%. Scale bar (a)–(i) = 5 *μ*m.

Electrospun fiber diameter distribution and morphology are shown in Figure 2. Fiber diameters were distributed between 50 and 1100 nm and were similar to PCL nanofibers containing DECM particles of all types as well as control fibers without particles. Fiber diameter distribution also appeared similar over the range of DECM particle concentrations applied (1%–10% w/w). PCL nanofibers meshes spun with DECM particles contained fibers with a beaded morphology in contrast to control fibers with a uniform fiber morphology. This beaded morphology was consistently present throughout all tissue types and concentrations. Safranin O staining of cartilage particles embedded in PCL nanofibers indicated that the DECM particles retained GAGs and that the nanoparticles may aggregate within the beaded regions of the nanofibers.

#### 3.2.2. Water Contact Angle

**Figure 3:**
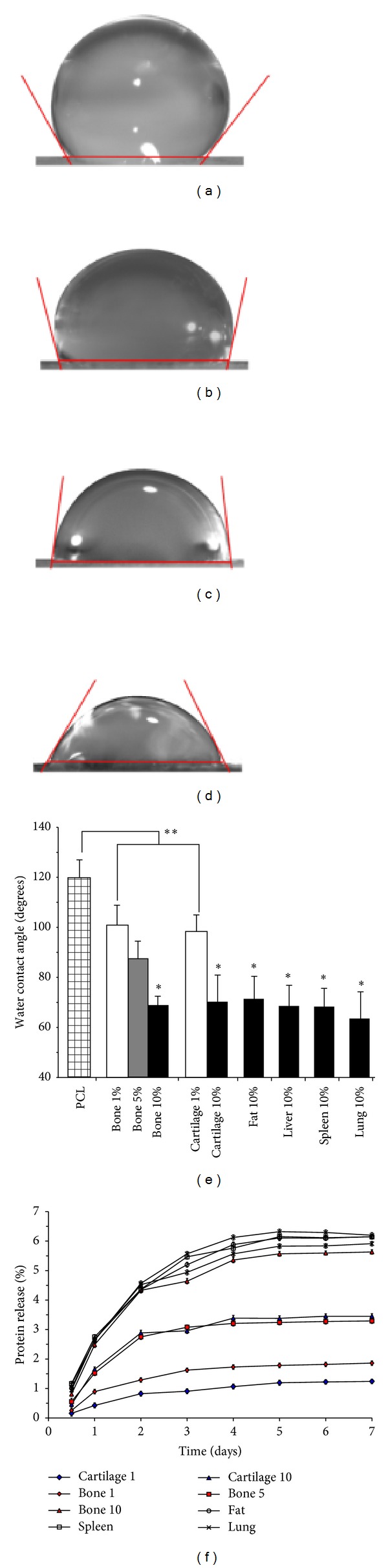
Representative image of water contact angles for nanofiber meshes consisting of PCL (a), 1% bone ECM (b), 5% bone ECM (c), and 10% bone (d). Water contact angles for each type of construct utilized in this study (e). Protein release into PBS over 21 days (f); *indicates *P* < 0.05 compared to fibers containing 1% ECM; **indicates *P* < 0.05 compared to PCL alone.

Results of water contact angle measurement on nanofiber meshes containing different DECM types and concentrations are shown in Figures 3(a)–3(e). A systematic drop in water contact angle was observed with increasing DECM particle concentration for all tissue types. Significant differences in contact angle were observed between particle concentrations of 0, 1, and 10%, but there were no significant differences in surface tension when comparing the different types of ECM at the same concentration.

#### 3.2.3. Protein Release

The release of proteins from PCL/DECM fiber meshes was characterized over time by monitoring protein concentration in incubated PBS for up to 21 days. The majority of protein was released in the first four days for all samples and the amount of protein released increased directly with increasing DECM particle concentration. An average of 5.53 (±1.08)% and 1.52 (±0.42)% of total protein was released within three weeks from fibers containing 10% and 1% ECM, respectively. The protein release profile (Figure 3(f)) was independent of the type of DECM contained in the fibers.

### 3.3. hASC Interactions with Composite Scaffolds

#### 3.3.1. Viability and Proliferation

**Figure 4:**
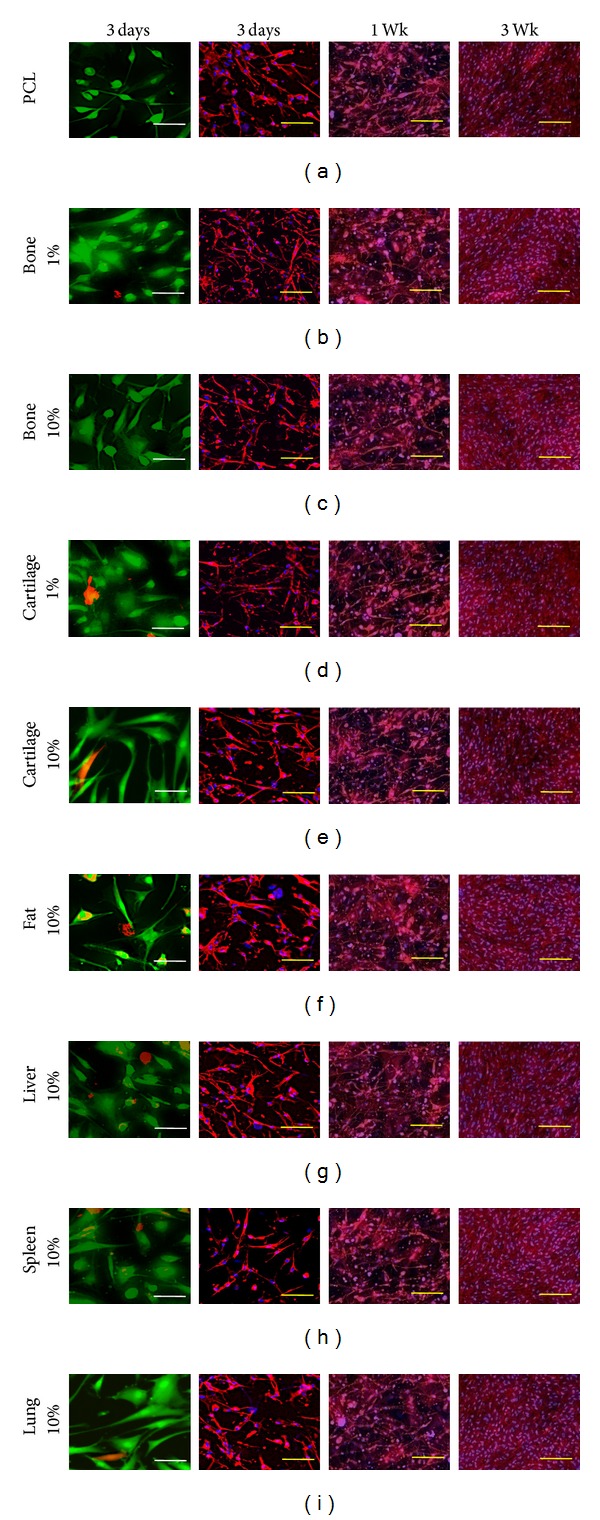
Viability and morphological structure of hASCs grown in monolayer on fibrous scaffolds containing ECM derived from the indicated tissues. In the left panel viable cells are stained green and dead cells are stained red with live/dead staining kit. In the middle and right panels actin is stained red (phalloidin, Texas Red) and cell nucleus is stained blue (DAPI). Scale bars for representative viability and morphology are 20 *μ*m and 100 *μ*m, respectively.

The viability and proliferation of hASCs cultured on each type of nanofiber scaffold were analyzed with fluorescent microscopy. Live/dead staining shows that hASCs attached and remained viable on nanofiber/DECM composites of all tissue types and concentrations (Figure 4). Images of hASCs with actin stained red and nuclei stained blue at 3 days, 1 week, and 3 weeks (Figure 4) show that cells proliferate and survive during long-term culture and become confluent on all composite scaffolds independent of tissue type and concentration. Observed cell morphology was relatively heterogeneous on all scaffold types at low densities and it became difficult to distinguish individual cells at high cell densities.

#### 3.3.2. Scaffold Effect on Osteogenic Differentiation

**Figure 5:**
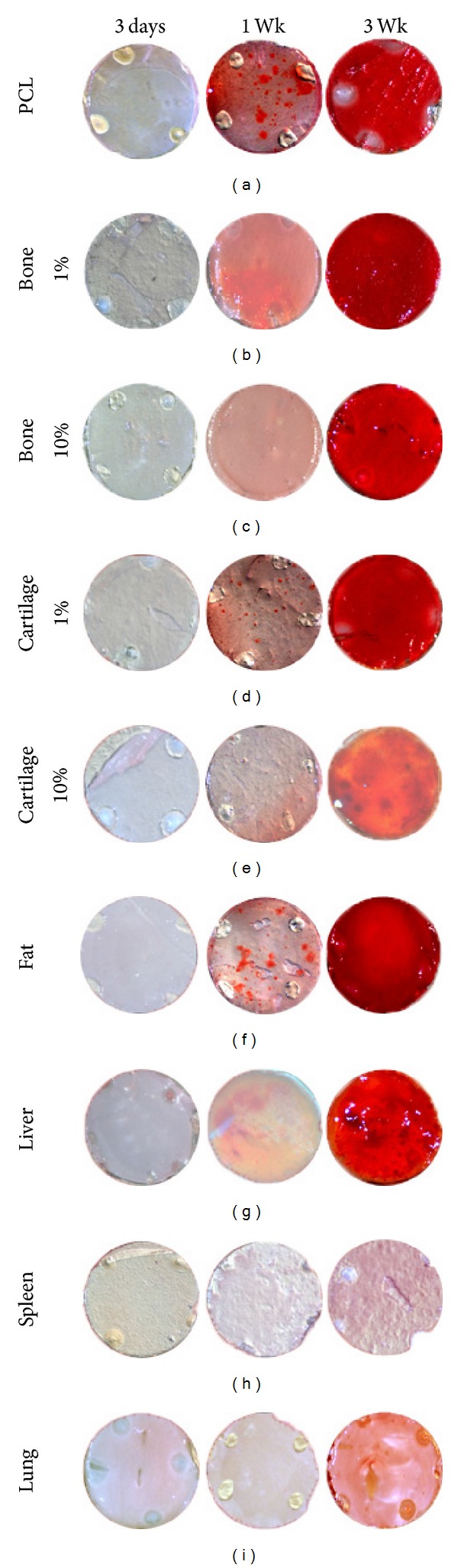
Alizarin red staining of hASCs in monolayer culture on fibrous scaffolds containing ECM derived from the indicated tissues. In each case, the diameter of the indicated circles is 5 mm.

The effects of DECM incorporation on hASC osteogenic differentiation were evaluated after 3 days, 1 week, and 3 weeks of culture on composite and control nanofiber scaffolds in osteogenic differentiation media. Visual comparison of calcified matrix production (stained red with alizarin red) in Figure 5 supports a relationship between scaffold DECM tissue type and osteogenesis. Control PCL scaffolds containing no DECM and composite scaffolds containing 1% bone, 10% bone, 1% cartilage, 10% fat, and 10% liver exhibited strong staining for mineralized matrix production after 3 weeks in culture (97.6, 97.4, 99.0, 97.1, 98.1, and 94.1%, resp.). In contrast, composite scaffolds containing 10% cartilage, 10% spleen, and 10% lung demonstrated very limited or no mineralized matrix production after 3-week culture (0, 0 and 1.1%, resp.).

**Figure 6:**
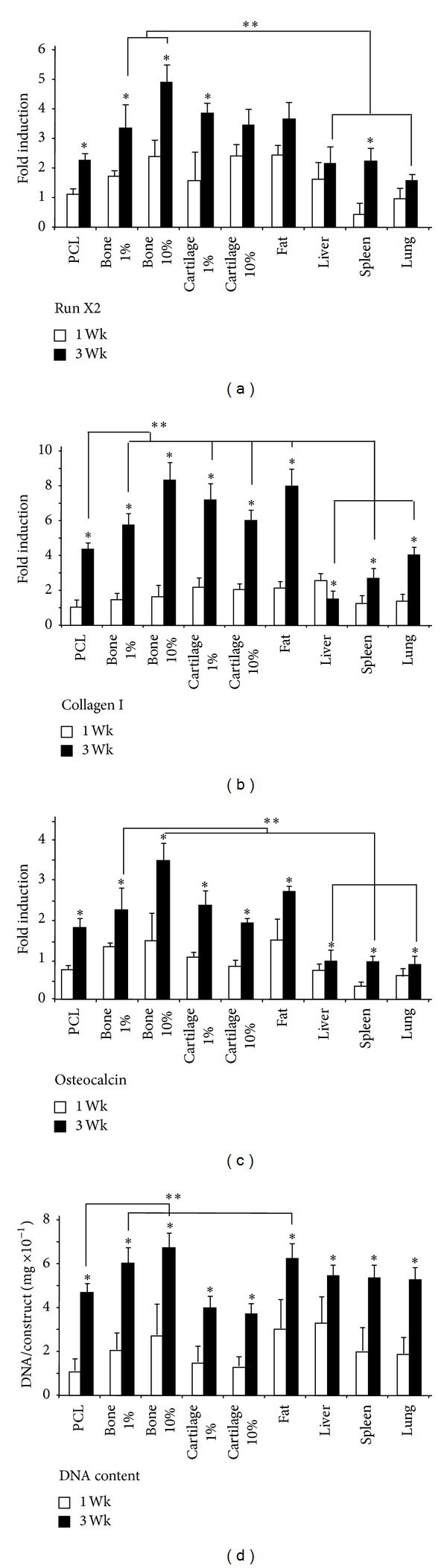
Gene expression of the osteogenic markers RunX2, osteocalcin, and collagen I following 1 week or 3 weeks of osteogenic monolayer culture on fibrous scaffolds containing the indicated ECM; *indicates *P* < 0.05 compared to 1-week time point for indicated ECM; **indicates *P* < 0.05 compared to 3-week PCL.

Expressions of genes commonly associated with hASC osteogenesis (RunX2, osteocalcin, and collagen I (col 1)) were compared with cells cultured for 1 week and 3 weeks on each type of nanofiber composite scaffold (Figure 6). Significant differences in osteogenic gene expression were apparent for all groups. RunX2, osteocalcin, and col I were upregulated in bone composites and showed a dose-dependent increase in expression when particle concentration is increased from 1% to 10% bone. All three osteogenic genes were also upregulated in scaffolds containing cartilage and fat, although, in contrast to bone scaffolds, osteogenic gene expression decreased when cartilage particle concentration was increased from 1% to 10%. Cells cultured on liver, spleen, and lung composite scaffolds all downregulated expression of RunX2, osteocalcin, and col I compared to control scaffolds.

## 4. Discussion

It is well established that decellularized ECM contains complex biological cues that retain the ability to influence cell function and behavior* in vitro* and* in vivo*. While DECM composition and its mechanisms of cell interactions remain incompletely understood, its value in regenerative medicine is underscored by the promising clinical performance of these materials. As previously discussed, DECM retains a complex structural and biochemical makeup that is highly bioactive. This bioactivity is difficult to replicate by adding purified biomolecules to scaffolds because of the vast assortment of biomolecules contained in DECM and their precise concentrations. Decellularized materials are also more cost effective and easier to implement from a regulatory perspective than many types of purified substances. The major limitations associated with DECM scaffolds are material properties, such as shape, mechanical strength, and scaffold degradation, which are difficult to control. These limitations are addressed by creating biosynthetic scaffolds that combine DECM with synthetic polymers and offer an alternative method of presenting DECM within a platform that allows tight control over scaffold material properties. The DECM/PCL nanofiber scaffolds presented here contain both insoluble biomolecules and soluble proteins that sustained extended release over several days. As cells break down the DECM component of these scaffolds, additional biomolecules may also be released from the scaffolds. The bioactivity of DECM/PCL nanofiber scaffolds was demonstrated by their tunable influence on osteogenic differentiation of stem cells.

Biomolecules may be incorporated into electrospun nanofibers by direct loading in a nanofiber solution prior to electrospinning or by subsequent surface modification techniques [16]. Aqueous insoluble molecules directly loaded into nanofibers may interact with cells at the fiber surface, and soluble biomolecules included within the nanofibers may exhibit controlled release into the surrounding environment. Incorporating biomolecules into nanofibers during electrospinning can be difficult, especially when the biomolecule's properties, such as solubility or charge, do not match well with those of the bulk polymer. Thus, problems such as low loading efficiency and burst release profiles are common [17–19]. Approaches designed to address these limitations are inclusion of carrier proteins, coaxial electrospinning, and micro-/nanoparticle loading [17, 20, 21].

In this study, decellularized tissue nanoparticles (<150 nm) were loaded into electrospun PCL nanofibers. Loading DECM in the form of nanoparticles presented several advantages. First, the DECM is left in a more natural state than solubilized DECM. Second, the nanoparticles can act as a reservoir for controlled release of proteins by desorption or cell-mediated degradation. Our results show that these composite nanofibers contain both insoluble and soluble biological components, whereas these factors would have to be added separately to an electrospinning solution using a purified substance approach. This is one example of how the complex makeup of DECM can fill multiple roles in scaffold design. A third advantage to using nanoparticles is that it eliminates the presence of additional solvents in the final electrospinning solution that may be required to maintain ECM solubility. ECM can be difficult to solubilize and different source tissues may require different types or concentrations of solvents. This adds a level of complexity to the electrospinning process, which is very sensitive to the composition of the final solution. Eliminating the need to solubilize DECM allows for a standard electrospinning approach that is compatible with many different sources of ECM.

Decellularized tissue nanoparticles were manufactured with a two-step method using a cryomill and emulsifier. This method of fabrication is advantageous in that it is repeatable and allows for very small particle size and uniform (±~100 nm) nanoparticle distribution. In addition, all of the different types of particularized tissue had similar size and size distribution profiles despite differences in structural and biochemical properties. Particles were incorporated into nanofiber scaffolds by simply adding them to a polymer solution prior to electrospinning. Nanofibers electrospun from solutions containing all types of DECM showed a beaded morphology commonly observed in electrospun scaffolds. Morphological similarity between scaffolds doped with different tissue types could be expected due to basic similarities in composition and particle size. The beads within the composite fibers were much larger than the size of the particles, so we hypothesized that they may be the result of agglomeration of tissue nanoparticles during the electrospinning process. Strong staining of cartilage particles in the region of the beads supports this hypothesis. Changes to the overall electrical properties of the polymer solution caused by the addition of the DECM particles could also contribute to the beaded fiber morphology. Quantification of protein release from the nanofibers proves that DECM is either contained on the nanofiber surface or able to diffuse through the PCL matrix. The presence of DECM on the nanofiber surface is further confirmed by the results of water contact angle testing. PCL is more hydrophobic than DECM so water contact angle decrease was used as a qualitative measure of DECM content on the nanofiber surface. Use of all types of DECM resulted in a significant dose-dependent decrease in water contact angle of composite nanofiber scaffolds. We attribute this reduction to increased scaffold hydrophilicity due to DECM content on the fiber surface and not morphological differences. Examples in the literature show that differences in morphology alone, such as the presence of beads, had a minor influence on water contact angle, while doping small intestine submucosa (SIS) into PCL nanofibers resulted in drops in water contact angle similar to our findings [22, 23].

Previous attempts to incorporate decellularized ECM into synthetic electrospun nanofibers are limited but have demonstrated promising* in vitro* and* in vivo* results. SIS/PCL nanofibers and urinary bladder matrix (UBM)/poly (ester-urethane) urea nanofibers both supported increased cell proliferation and adhesion* in vitro* compared to synthetic controls [24, 25]. Nanofiber scaffolds containing UBM also resulted in increased degradation and cell infiltration* in vivo* compared to controls [24]. UBM was included in an electrospinning solution after pepsin digestion and SIS was added to PCL solution as a heterogeneous powder sieved below 2 *μ*m. The present study expands the versatility of DECM electrospinning by developing a consistent methodology to form nanoscale particles with much smaller size (~100 nm diameter) and a narrow size distribution. Furthermore, particles with similar size and distribution profiles could be formed from tissues with varied compositions and mechanical properties without altering the procedures. Because these nanoparticles are small enough to be embedded within electrospun nanofibers, tissue type selection is not limited by solubility issues associated with electrospinning and virtually any source tissue should be compatible. Thus, this method represents a versatile platform in which different tissue types or combinations of tissues can be included in a nanofibrous biosynthetic scaffold based on the desired bioactivity for a specific application. The importance of this versatility was demonstrated by showing the unique effects that each tissue type had on the osteogenic differentiation of hASC cells.

Composite DECM/PCL nanofiber scaffolds demonstrated the ability to modulate osteogenic differentiation of hASC cells according to tissue type and concentration. Osteogenesis was determined by measuring stem cell tissue production and gene expression* in vitro*. Both methods supported the capacity of DECM to influence the differentiation of hASC cells cultured on composite scaffolds. Some discrepancies between tissue production and gene expression were observed such as higher alizarin red staining for liver compared to lung and spleen groups despite similar osteogenic gene expression at week 3. We hypothesize that this could be because gene expression is a snapshot while calcified matrix production is collective, so calcium matrix production between day 1 and week 3 may have been much higher on liver. ASC cells on spleen may still be in an early differentiation stage at week 3 where RunX2 and osteocalcin are upregulated, but calcified matrix production has not begun or has just recently begun near the 3-week time point and deposition is not adequate for positive staining. Another variable that could affect the timing of osteogenic gene expression and calcified matrix production is the degradability of DECM which would influence biomolecule release profiles and could render the effects of any DECM to be short term.

Unfortunately, it remains difficult to determine the mechanisms by which composite DECM/PCL scaffolds affect osteogenesis. Morphological differences in nanofiber scaffolds have been shown to affect cell shape and cell shape, in turn, has been implicated in stem cell differentiation. Thus, morphological differences in scaffold types could have had an impact on the osteoinduction of hASCs in this study. However, cell shape changes related to nanofiber morphology are commonly associated with order of magnitude differences in fiber diameter, and cell shape changes associated with the differentiation path appear to be quite noticeable in the literature [26–28]. Differences in fiber and hASC cell morphology among different scaffold types appeared to be limited in these studies, so we expect that cell interactions with the tissue-specific DECM component of the scaffolds played the primary role in stimulating or impeding osteogenesis.

DECM nanoparticles may be recognized by integrin receptors or potentially could be extracted from the fiber matrix and endocytosed by the cells. Thus, while protein release measurements confirm the presence of DECM nanoparticles in the PCL scaffolds, cells attached to the scaffolds may experience higher concentrations of protein due to integrin binding, endocytosis, and local protein release by cell mediated degradation. Specific interactions of the cells with the particles could have an effect on gene expression and on protein release into the media if the ECM is broken down by attached cells enzymatically. We hypothesize that interactions with specific biomolecules contained in the tissue ECMs are modulating osteogenic differentiation and that the variation amongst different tissue types is because they have different biochemical compositions. In future studies, characterization techniques such as immunohistochemistry or proteomic analysis with mass spectrometry [29] could be used to identify specific bioactive components contained in the DECM nanoparticles. Perhaps, biomolecules inhibitory to osteogenic differentiation and matrix calcification such as matrix GLA protein, osteoprotegerin, or FGF [30] are contained at higher levels in tissues, such as lung and spleen, which inhibited calcified matrix deposition. Relative increases in osteogenesis associated with tissues such as bone and fat could be driven by higher content of embedded factors that promote osteogenesis such as bone morphogenetic proteins (BMPs) [31] or osteogenic favorable combinations and ratios of ECM proteins such as laminin and fibronectin [32, 33]. Alternatively, biomolecules contained in the ECMs may indirectly affect matrix calcification by activating other processes. For example, an immune response could trigger ASC cells to secrete cytokines or growth factors [34] that subsequently play a role in stem cell differentiation such as TGF-*β* [35].

The exact composition of the DECM is not fully understood; therefore, its mechanisms of interaction with cells are even more difficult to determine. However, much research is currently being undertaken to learn more about the proteomic composition and* in vitro* and* in vivo* cellular interactions with decellularized scaffolds. Here, biosynthetic DECM/PCL nanofiber scaffolds were able to sharply influence the osteogenesis of hASC cells; this effect was strongly related to tissue type and demonstrated dose dependence. While the precise mechanism of action remains unknown, the bioactivity of these composite scaffolds may improve clinical outcomes. Logically, composite scaffolds containing bone particles demonstrated the highest level of osteogenic gene upregulation and, thus, would be obvious candidates for bone regeneration applications. Perhaps even more interestingly, the varied effects of different tissue types on hASC osteogenesis highlight the potential use of these DECM/polymer nanofiber composites in a wide variety of applications for which the DECM tissue type, or combination of types, can be selected based on the desired application. One example where this scaffold customizability would be useful is in applications where bone formation is undesirable, such as craniosynostosis. Here, a composite nanofiber scaffold containing spleen DECM may impede undesirable bone formation, as indicated by its* in vitro* interactions with hASC cells. In a broad view of these biosynthetic scaffolds, the polymer nanofiber component serves as a primary structural component and the DECM nanoparticles serve as a modular component selected based on the desired bioactivity for a specific application.

## 5. Conclusions

The research presented here includes an effective and versatile method of incorporating decellularized ECM into biosynthetic scaffolds and demonstrates the differential effects that different tissue types can have on osteogenesis within the context of these scaffolds. Decellularized ECM from bone, fat, cartilage, liver, lung, and spleen tissue was incorporated into a biosynthetic nanofiber composite scaffold as nanoparticles (~100 nm) with narrow diameter distributions. A major advantage of these nanofiber composites is the ease of interchangeability of different types of DECM within the scaffolds. Despite differences in DECM tissue type, composite scaffolds demonstrated similar morphological properties and facilitated similar cell viability and morphology* in vitro*. However, osteogenesis of hASC was differentially promoted or impeded depending on the tissue of origin of the DECM contained in each scaffold. Given the inherent bioactivity of the DECM nanoparticles and the versatility of the fabrication method, we hypothesis that biosynthetic scaffolds with customizable bioactivity can be fabricated within this platform.
